# Neuroendoscopy-compatible neurostimulation catheter for minimally-invasive and multifunctional hypothalamic deep brain stimulation

**DOI:** 10.1007/s10544-026-00804-2

**Published:** 2026-03-12

**Authors:** Jae Young Park, Juan C. Mesa, Jongcheon Lim, Deniz Eksioglu, Sergio Ruiz Vega, Albert Lee, Hyowon Lee

**Affiliations:** 1 Weldon School of Biomedical Engineering, Purdue University, West Lafayette, IN USA; 2 Birck Nanotechnology Center, Purdue University, West Lafayette, IN USA; 3 Center for Implantable Devices, Purdue University, West Lafayette, IN USA; 4 Legacy Health, Portland, OR 98671 USA

**Keywords:** Biomedical microdevice, Implantable medical device, Neuroengineering, Neural interface, Deep brain stimulation, Neurosurgery

## Abstract

As hypothalamic deep brain stimulation (DBS) has been clinically proven effective for treating various neurological conditions, there is a growing need for strategies that minimize invasiveness while maximizing therapeutic efficacy. We propose a highly translational multifunctional neurostimulation catheter compatible with endoscopic access to the third ventricule for targeted ventromedial hypothalamic deep brain stimulation. This approach offers several key advantages: (1) a multifunctional catheter for both electrical and pharmaceutical intervention, (2) a minimally-invasive implantation procedure, and (3) enhanced electrode contacts with the third ventricle wall to access the underlying hypothalamic nuclei. The system comprises a flexible catheter with integrated thin-film microelectrodes, a customized cylindrical connector, extension wires, and a customized neurostimulator. The design features its high compatibility with clinically available neurosurgical tools, including guide wires and burr hole valves. We evaluated electrochemical performance under various bending conditions and assessed the safety and long-term device reliability. In addition, we demonstrated successful implantation into a Polydimethylsiloxane (PDMS) mold shaped to sit in the third ventricle, simulated the electric potential distribution using finite element analysis, and validated a clinically compatible drug delivery procedure. This novel implantation strategy holds promise for reducing procedural risks associated with hypothalamic deep brain stimulation.

**Supplementary Information:**

The online version contains supplementary material available at 10.1007/s10544-026-00804-2.

## Introduction

Deep brain stimulation (DBS) is an invasive procedure of placing stimulating electrodes in a certain nucleus of the brain and periodically delivering variable frequency electrical pulses. Electric fields generated by the metal contacts activate the white matter neurons triggering action potentials to propagate (McIntyre et al. 2004), with varying multiscale mechanisms based on the therapeutic objective. Subthalamic nucleus and Globus Pallidus Internus has been the primary target for treating Parkinson’s disease for a long time (Okun 2012), but recently, other nuclei, such as subgenual cingulate cortex, nucleus accumbens, and hippocampus, are being explored for the treatment of neurological conditions such as depression, obesity, compulsive disorders, and Alzheimer’s disease (Lozano et al. 2019).

**Fig. 1:**
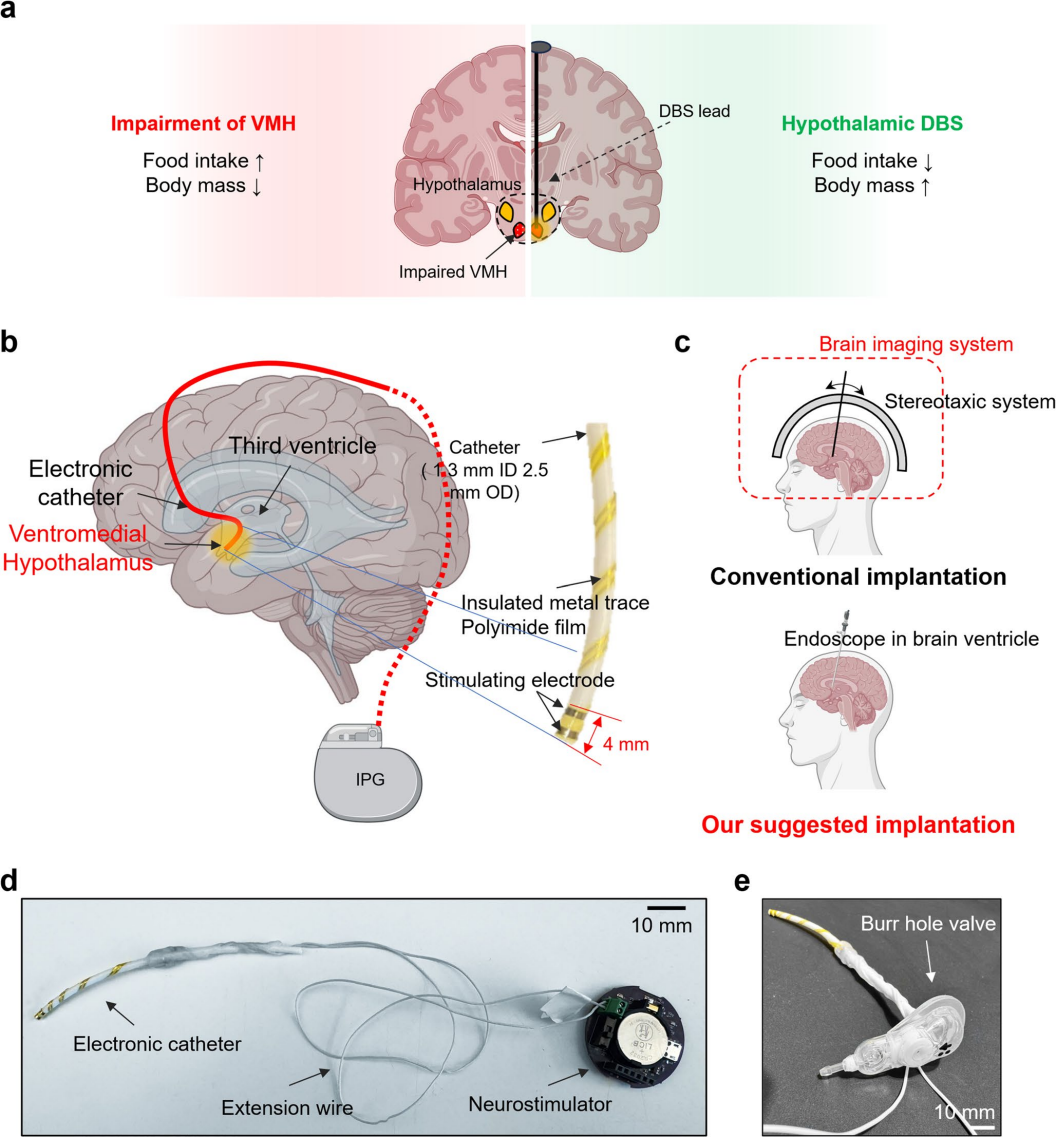
**a** Therapeutic effect of stimulating ventromedial hypothalamus for treating morbid obesity. **b** Overview of the neuroendoscopy-compatible neurostimulation catheter for hypothalamic deep brain stimulation. **c** Comparison between the conventional DBS surgical procedure and the neuroendoscopy-guided DBS implantation. **d** Photograph of fully-integrated electronic catheter system **e** neurostimulation catheter covered by burr hole valve for drug delivery application

Of particular interest is the growing interest in applying DBS for treating morbid obesity. Obesity rates have more than doubled globally since 1980, affecting over 600 million adults and 100 million children by 2015, and are closely associated with various serious health conditions that worsen mortality and life quality (GBD 2015 Obesity Collaborators 2017). Especially, in the case of obese or severely obese, lifespan may drastically reduce, ranging 5.6–10.3 years (Lung et al. 2019). For a long time, considerable effort has been dedicated to understanding the functional roles of the hypothalamus in regulating food intake (Blouet et al. 2009; Yousefvand and Hamidi 2021). Strong evidence suggested that the ventromedial hypothalamus (VMH) functions as a satiety center in the central nervous system: early studies involving non-selective physical damage or chemically-induced impairment of the VMH have led to excessive food intake (Balagura and Devenport 1970; Becker and Kissileff 1974). Moreover, several recent studies have demonstrated that food intake is controllable through stimulating the ventromedial hypothalamic region (Lehmkuhle et al. 2010; Melega et al. 2012) (Fig. 1a), which is also being extensively investigated for clinical application (Torres et al. 2013; Salles et al. 2018). Although many useful options for treating morbid obesity, such as pharmacotherapy and bariatric surgery, are available, the long-term safety and efficacy remain unclear (Formolo et al. 2019).

The process of implanting a DBS lead requires the highest level of precision. It’s a complex invasive task that calls for experienced neurosurgeons and specialized equipment, including stereotactic and imaging systems, to penetrate through the brain tissue. Despite these precautions, electrode misalignment remains a critical challenge (Pearce et al. 2021). It has been reported that the implanted electrode can shift up to 6 mm^15^ by brain movement from air invasion in the subdural space and the loss of cerebrospinal fluid during the surgery (Contarino et al. 2013). Also, other causes such as variations in surgical technique, lead fixation failure, Twiddler syndrome, and pneumocephalus can lead to further displacement of the electrode (Göransson et al. 2021). Any misalignment can lead to off-target effects that could compromise the efficacy of the treatment (Zarzycki and Domitrz 2020). In severe cases, the misalignment necessitates the removal of the electrode, and the patient must undergo a second surgery, which further increases the risk of permanent injury.

Seeking a solution to these challenges, such as steerable and selective neural stimulation (Lim et al. 2024; Lehto et al. 2017), a group of neurosurgeons proposed an alternative route for DBS insertion, specifically through the third ventricle (Chabardès et al. 2016). This approach takes advantage of the fact that the ventricle’s wall is adjacent to the hypothalamus. However, it presents its own set of obstacles. Achieving ideal electrode contact, which is crucial for reliable stimulation, has proven to be difficult. Moreover, this approach requires the use of state-of-the-art, highly expensive equipment, including a stereotactic navigation that at times uses robotic targeting, fluoroscopy or other intraoperative imaging systems to ensure the necessary level of precision.

To overcome these limitations, here we propose to use an endoscopic approach to the third ventricle as an alternative way to access the third ventricle for the implantation using our bespoke catheter-based neurostimulation interface (Fig. 1b). As illustrated in Fig. 1c, this methodology utilizes a standard neuroendoscopic setup, equipment that is not typically employed in conventional DBS procedures. The ventricular walls can contract and expand around the catheter because the diameter of the catheter is larger than conventional DBS leads. In this work, we have developed a novel and highly translational neurostimulation catheter that is compatible with an endoscopic approach to third ventricle by integrating stretchable electrodes onto a flexible silicone catheter. The design maintains high compliance with the original silicone catheter by minimizing changes in its mechanical properties and form factors after integration (Fig. 1d). Furthermore, the catheter’s inner lumen and compatibility with standard ventricular access devices, such as ventriculoperitoneal shunt products (Fig. 1e). By leveraging a simplified surgical approach and integrating multifunctionality into our platform, this system offers a more precise method for DBS lead placement with increased effective therapy for hypothalamic stimulation. Although translating implantable microsystems into clinical use is highly challenging (Park et al. 2025), the system’s strong compatibility with clinically available protocols and neurosurgical components brings it closer to clinical application.

## Results and discussion

### Microfabricated electronic catheter for third ventricle implantation

To be compatible with neuroendoscopy, the microfabricated device was designed as a catheter with neurostimulation microelectrodes for deep brain stimulation. Compared to the conventional DBS leads, which are stiff and unable to make intimate contact with the third ventricle wall, a silicone-based ventricular catheter is ultra-flexible and fits in the ventricle system according to the previous study for evaluating the self-clearing catheter in lateral ventricle done by our group (Yang et al. 2022). In addition, its outer diameter (2.5 mm) is larger than that of commercial DBS leads to ensure an intimate interface when the ventricle shrinks after the surgery. The inner diameter of the catheter (1.3 mm) is larger than the diameter of a flexible optical fiber endoscope (0.8 mm) to contain it inside the catheter for third ventricle navigation. For this reason, we have adapted a new design of a thin-film electronic device-wrapped silicone catheter structure.

**Fig. 2:**
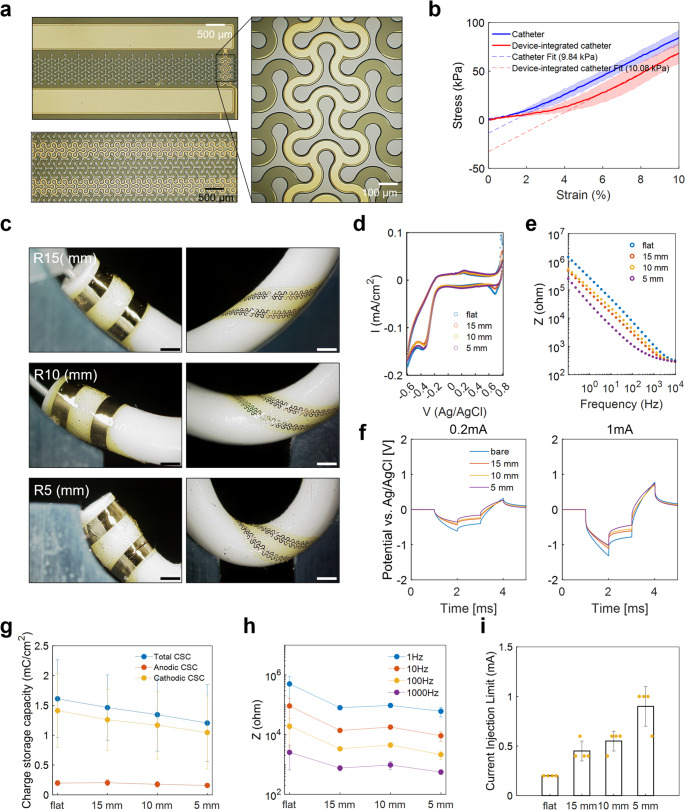
**a** Photographs of thin-film electrodes with kirigami-patterned traces. **b** Comparison of Young’s modulus between a bare silicone catheter and the device-integrated catheter. **c** Photographs of electronic catheter in various bending radius: 15 mm, 10 mm, 5 mm. **d** Cyclic voltammetry **e** electrochemical impedance spectroscopy. **f** Voltage transient response **g** charge storage capacity. **h** Impedance at specific frequencies. **i** Current injection limit according to various bending radius

The thin-film device was microfabricated using conventional MEMS technologies on a 4-inch silicon wafer (Fig. S1a). Gold, which is one of the renowned electrode materials for neuromodulation, is deposited on the neutral plane of a 10 μm-thick polyimide film as illustrated in Fig. S1b. The stimulating electrode part was horizontally attached to the catheter while the trace part was spirally wrapped around it. Silicone glue (DOWSIL 734) was used to give adhesion between the device and the catheter, which requires 1 day of incubation at room temperature. To avoid any stress localization or kink of the device according to the catheter’s bending, we endowed stretchability on the device with void microstructures of Y-shaped Kirigami pattern (Vachicouras et al. 2019) (Fig. 2a), which was explored by our group through integration with a hydrogel adhesive for vagus nerve stimulation (Park et al. 2025). As shown in Fig. S2d, the kirigami-shaped trace showed strain-insensitive resistance, which didn’t increase up to 2 times of initial value until it failed. Because our Kirigami-structured device is highly stretchable and conformal to a silicone catheter, as shown in Fig. 2b, the Young’s modulus didn’t increase significantly from that of the silicone catheter (~ 2% increase). The result shows that the addition of thin-film electronics does not alter or complicate the endoscopic surgical procedure, which is critical for clinical adoption.

### Electrochemical characterization depending on the bending radius

**Fig. 3:**
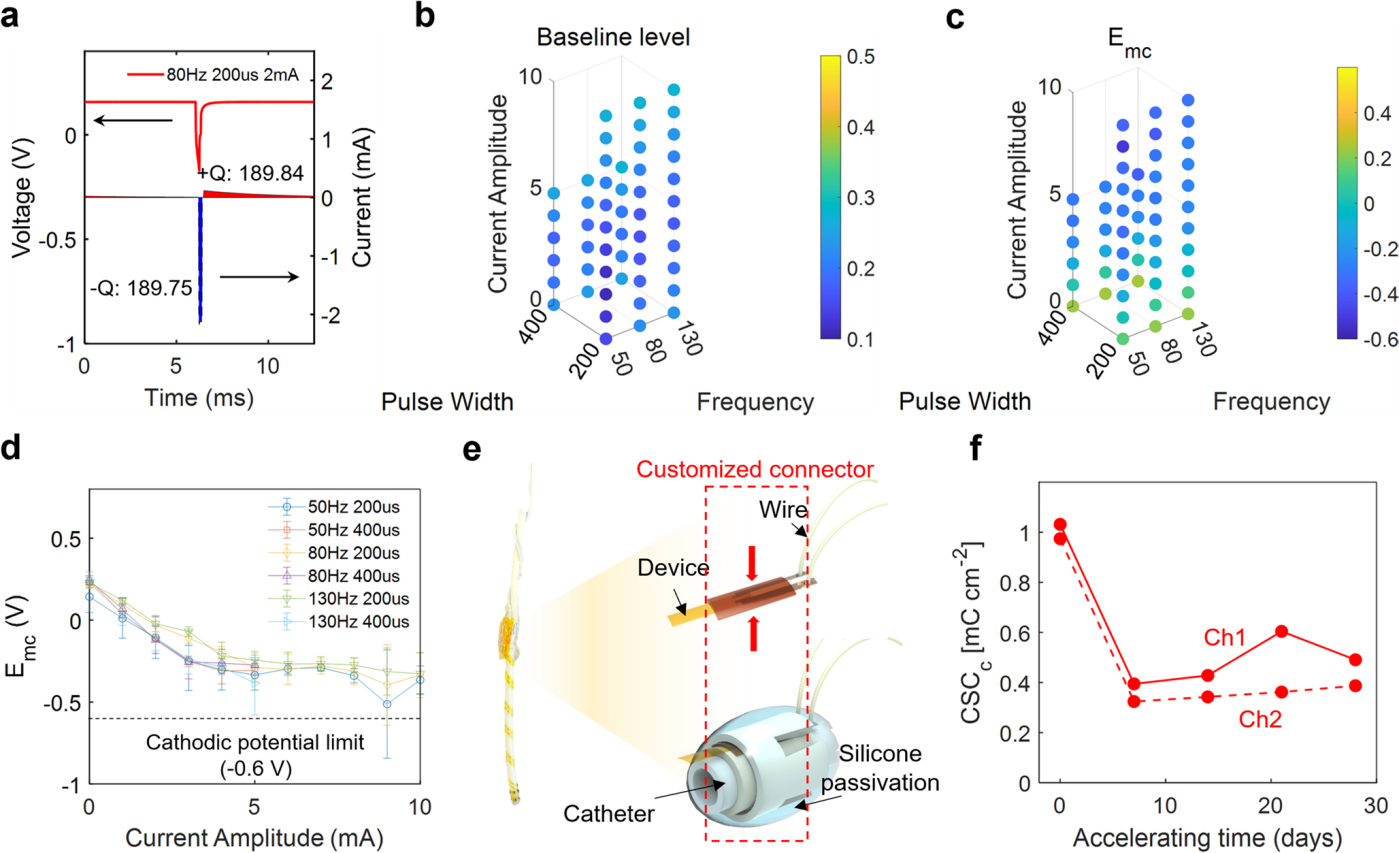
**a** Injected current waveform and corresponding voltage transient from the electrode. **b** Baseline current level and **c–d** Negative excursion potential under various stimulus parameters. **e** Customized connector interfacing the device with silicone-insulated wires **f** Results from an accelerated aging test at 67 °C for 4 weeks

Next, the bending radius-dependent electrochemical properties were characterized. To place the catheter in the third ventricle, the tip was bent inside to allow the catheter to navigate the curvature of ventricle pathway. As such, it is important to verify that the neurostimulation electrodes on catheter maintain electrochemical functionality when it is bent or twisted in situ. Cyclic voltammetry (CV), Electrochemical impedance spectroscopy (EIS), and Voltage transient response (VT) were measured in a controlled manner for bending radius with a 3D printed fixture. Figure 3c shows that the DBS traces closely conform to the curved surface of the bent catheter, enabled by the enhanced stretchability by the kirigami microstructures. A radius of 5 mm was considered as the extreme bending condition based on a previous study on mechanical testing of DBS lead (Jiang et al. 2015). From CV results as shown in Fig. 2d, the values of charge storage capacity (CSC), which is one of the predictors of charge injection capability (Hudak et al. 2017), were calculated for all conditions (Fig. 2g). In Fig. 2d, the catheter under bending conditions exhibited a reduced cathodic peak compared to the unbent state, although the dependence on bending radius was negligible. However, some samples showed a pronounced tendency according to bending radius (Fig. S3), suggesting that the extent of this effect depends on how tightly the device is integrated with the catheter. Electrochemical impedance spectroscopy (EIS) was also measured, and the impedance values at specific frequencies (Fig. 2h) were extracted to observe the bending radius-dependent changes. As the bending radius increased, the impedance gradually decreased across the entire frequency range (e.g. 503 KΩ ~ 61 KΩ at 1 Hz, 193 KΩ ~ 2 KΩ at 1 kHz) due to a progressive shift in the transition frequency, suggesting an enhancement of the electrical double layer in the deformed electrodes. Moreover, negative excursion potential, current injection limit (Fig. 2i), and load energy are calculated from VT results as shown in Fig. S4d. Negative excursion potential and current injection limit indicate the maximum current amplitude for electrode safety and load energy refers to power consumption during stimulation. Figure 2f shows that the voltage transient during cathodic charge injection is primarily influenced by the bending effect, which leads to an increased current injection limit up to 0.9 ± 0.2 mA (Fig. 2i) and a reduction in load energy (Fig. S4d). All the electrochemical results show that the electrode is functional in all the bending radius variables up to 5 mm. Even in most results except CSC, the capability is improved as the bending radius decreases.

### Stimulation capacity in artificial cerebrospinal fluid

Knowing the electrode’s current injection limit in the actual environment (cerebrospinal fluid, CSF) is crucial in avoiding electrode dissolution with electrolysis. For this reason, artificial CSF (aCSF) was prepared as a medium for characterizing the device. As shown in Fig. S5, there was no significant difference between 1x PBS and aCSF in CV and EIS. Voltage transient response was also systematically investigated depending on multiple parameters, including pulse width, frequency, and current amplitude of the input pulses, as shown in Figs. 3a–c. Figure 3a shows the charge-balanced biphasic current pulse delivered to the electrode and the corresponding voltage transient response in one of the stimulus parameters utilized. The ranges of pulse width, frequency, and current amplitude were selected based on previous studies investigating stimulus parameters for hypothalamic deep brain stimulation. The maximum stimulus parameter was decided based on 30 µC/cm^2^/phase, which is reported as a maximum charge density per phase for safety (Melega et al. 2012). The asymmetric biphasic pulse was injected into the bipolar electrodes of the anode and the cathode from an implantable neurostimulator. Because the negative excursion potential is also influenced by the voltage baseline, which refers to electrode polarization, it is important to investigate the baseline level according to multiple parameters. According to Figs. 3b and S7, the baseline voltage in aCSF was approximately 0.2 V, whereas in PBS it was around 0.4 V (Figs. S8–S9), indicating that the composition of physiological ions influences electrode polarization. In Fig. 3d, the negative excursion potential became more negative with increasing current amplitude, but did not reach the cathodic limit of -0.6 V. This indicates that all the stimulus parameters used remained within the device’s charge injection limit.

To ensure the entire catheter fits within the ventricle route, we designed a customized cylindrical connector that bridges the thin-film pads and silicone-insulated wires without using bulky components. After soldering the wires to the metal traces, they were mechanically secured to the thin-film metal pads by tightening them between two interlocking 3D-printed cylindrical components (Fig. 3e). This approach eliminates the need for adhesives, which could swell under high temperature or humidity potentially compromising conductivity (Mercado et al. 2003). Nevertheless, exposing the connector can introduce critical issues: it may induce off-target effects and trigger toxic reactions with the physiological environment due to the lead component from the soldering process. To ensure proper passivation, a sufficiently thick layer of silicone glue was applied to cover the entire connector area.

### Device integration and in vitro demonstration

**Fig. 4:**
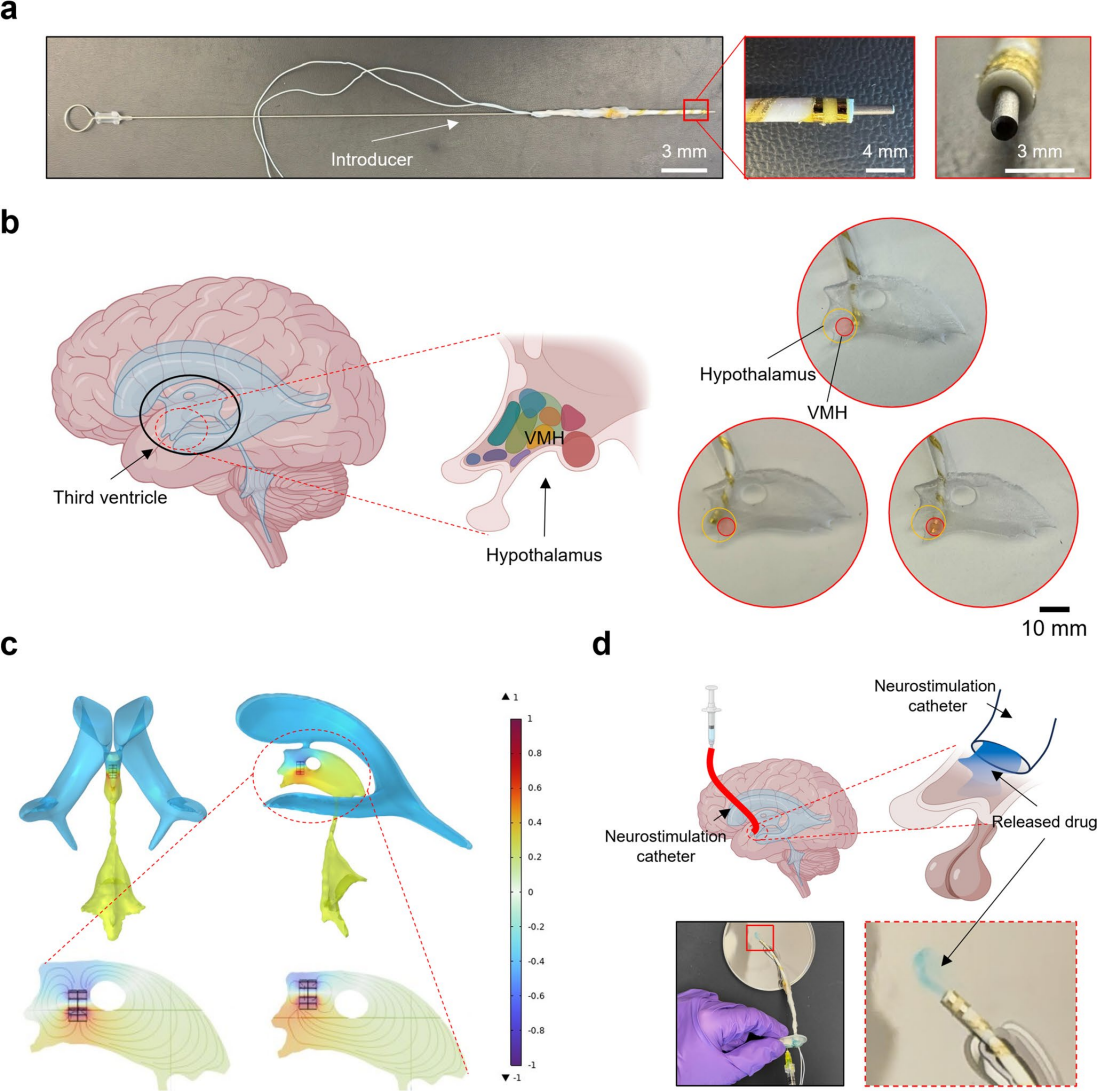
**a** Photographs of compatibility with neurosurgical introducer. **b** Demonstration of neurostimulation catheter implantation into the third ventricle using a PDMS mold. **c** Simulated electric field distribution generated by two opposite potentials applied to the anode and cathode of the device. **d** Demonstration of drug delivery application through burr hole valve

After evaluating the charge injection capability of the assembled lead, its compatibility with the neurosurgical procedure was assessed using a benchtop model and FEA simulation. Figure 4a shows that the inner diameter of the electronic catheter closely matches the dimension of the neurosurgical introducer, demonstrating its suitability for the endoscopic procedure. In addition, to evaluate whether the electronic catheter can be stably positioned within the third ventricle and maintain effective contact with hypothalamic tissue, a Polydimethylsiloxane (PDMS)-based model replicating the third ventricle was fabricated, as shown in Fig. 4b. To insert the catheter without damaging surrounding tissue, the ventricle needed to be first dilated by injecting artificial cerebrospinal fluid. After proper placement of the device, the fluid was extracted to allow the ventricle to return to its original shape. The natural constriction of the third ventricle walls and the foramen of Monro helped anchor the device and minimize the risk of migration. Because the walls of the third ventricle narrow toward the bottom, positioning the catheter tip at the base further enhances implantation stability. Moreover, we explored targeted stimulation of the ventromedial nucleus in the hypothalamus, a promising site for hypothalamic DBS (Formolo et al. 2019), by simulating various catheter placement strategies within the third ventricle model. Depending on the bending state of the catheter, the contact location with the ventricular wall varied (Fig. 4b), which may influence the extent of the therapeutic effect. Finite element analysis (FEA) was used to simulate the electric potential distribution within the fluid-filled ventricle during stimulation. As shown in Fig. 4c, when a 1 V was applied to stimulating electrodes, regions throughout the ventricle, including the most distal areas, were biased up to around 0.2 V for anodically-affected region and − 0.43 V for cathodically-affected region due to the conductivity of the surrounding fluid, indicating that the entire ventricle space can be influenced during stimulation,. Although the high conductivity of CSF allows hypothalamic DBS without requiring ideal electrode contact, as shown in previous study (Chabardès et al. 2016), further analysis is needed to better understand the specific volume of the targeted area in this conductive environment. A magnified view of the third ventricle region reveals that the underlying neutral substrate is activated by neurostimulation varied with the catheter’s position. These results highlight the importance of anatomically guided placement of the catheter to ensure consistent therapeutic outcomes. Furthermore, the demonstration of cerebrospinal fluid accessibility by integrating the device with a shunt hole valve (Strata™ Burr Hole Valve, Medtronic, USA) or drug delivery via an Ommaya reseiboir (Fig. 4d), highlights its multifunctionality for both electrical and chemical treatments.

## Discussion

In this work, we developed a novel neurostimulation catheter designed for implantation in the third ventricle to enable endoscopic surgical placement of a multifunctional hypothalamic deep brain stimulation. Benchtop experiments validated not only its bending-dependent electrochemical behavior and safety considerations but also its strong compatibility with clinical neurosurgical components for implantation and drug delivery procedures. Additionally, both experimental and finite element analysis-based demonstrations of implantation in the third ventricle confirmed the feasibility of the approach. Despite these promising results, several limitations in this study must be addressed to advance this proof-of-concept toward clinical translation. First, while our benchtop experiments demonstrated basic electrochemical stability, rigorous biocompatibility testing according to international standards, such as ISO 10993-1, is mandatory to ensure the long-term safety of the catheter materials within the ventricular environment. Second, in vivo studies are required to validate whether the surgical procedure is compatible with the complex anatomy of a living brain and to evaluate the extent of the foreign body response triggered by the implant. Finally, chronic stimulation tests are essential to confirm the long-term therapeutic efficacy of this approach for treating morbid obesity. Such studies will be crucial for monitoring potential adverse effects caused by off-target stimulation. Should these issues arise, further optimization of stimulus parameter will be necessary to maximize the therapeutic window and ensure precise targeting of the ventromedial hypothalamus.

## Methods

### Device fabrication

The device fabrication procedure follows the approach outlined by Lim et al. (2023). The procedure began with spin-coating a 5-µm-thick polyimide (PI, PI2545, HD Microsystems, Parlin, NJ) layer onto a 4-inch silicon wafer, followed by thermal curing at 300 °C for 1 h under a nitrogen flow. Photolithographic patterning was carried out using a positive photoresist (PR, AZ9260, MicroChem, Newton, MA, USA) and a maskless photoaligner (MLA150, Heidelberg Instruments, Germany). The exposed samples were developed by submerging them in a diluted developer solution (AZ400K: DI = 1:3), followed by a thorough rinse with deionized (DI) water. Next, a 10 nm-thick titanium layer and a 100 nm-thick platinum layer were sequentially deposited using sputtering, after which a lift-off process was carried out. To create a passivation layer, a second PI layer, 5 μm thick, was deposited using the same procedure as the bottom PI layer. The second photolithography step was then conducted on a double-coated PR barrier, which resulted in a total thickness of 10 μm, to expose the stimulating electrodes and contact pads. The patterns were etched using a reactive ion etch (100 W, 50 sccm O_2_, 50 mTorr). The same photolithography and RIE procedures were repeated to pattern the outline of the device. Finally, the device was detached from the wafer by removing the native oxide layer with buffered oxide etch and thoroughly rinsed with DI water.

### Electrochemical characterization

Potentiostat (SP-200, BioLogic Inc., Seyssinet-Pariset, France) was used to perform cyclic voltammetry (CV) and electrochemical impedance spectroscopy (EIS) in a three-electrodes setup within a 1X phosphate-buffered saline (PBS) solution (Thermofisher Scientific, Waltham, MA, USA). The scanning range for CV was between − 0.6 V and 0.6 V versus Ag/AgCl at a scan rate of 50 mV/s. The sweeping frequencies in EIS ranged from 10 kHz to 0.1 Hz where the sine wave is 30 mV relative to open circuit potential.

### Voltage transient analysis

Voltage transient (VT) measurements were performed to investigate the charge injection limit (CIL) of the Kirigami electrodes, using a Howland current pump circuit-based stimulator. A custom MATLAB-based stimulation software developed by Ward et al. (2015) was used to generate cathodic-first biphasic symmetric current pulses through an I/O data acquisition board (USB-6361, National Instruments, Austin, TX, USA). The stimulus parameters were determined based on previous study (Lim et al. 2023), with a frequency of 50 Hz, a pulse width of 0.5 ms, and a current amplitude ranging from 0 to 1 mA, applied over a 1-second duration. The simulation was applied between the kirigami electrode and a counter electrode (graphite), while the voltage response was recorded in real-time between the stimulating electrode and a reference electrode (Ag/AgCl).

### Mechanical test

To evaluate the Young’s modulus, a tensile test was conducted using universal testing machine (SM-250-294, Test Resources Inc., Shakopee, MN, USA). Based on ASTM D638-14. The tensile strain rate was determined to be 50 mm min^− 1^. To characterize strain-dependent resistance, we conducted an electromechanical test by applying a 10 V across the metal trace while monitoring the current to determine its resistance response under tensile strain. A tensile tester (Mark-10, Copiague, NY, USA) was used to apply the strain, with data acquisition synchronized between the tensile tester and resistance measurements via a customized LabView interface (National Instruments, Austin, TX, USA). For electrical connections, silver conductive epoxy (8331D-A, MG Chemicals, Ontario, CA, USA) was applied to L-shaped connector pins, which were carefully positioned on the specimen’s contact pads. The connections were then encapsulated with a silicone adhesive (DC 734 RTV clear, Dow Corning, Midland, MI, USA).

### Ventricle model-based studies

The third ventricle model used for demonstration and simulation was created by Thingiverse user akshay_d21, and is licensed under Creative Commons (CC) licenses: https://www.thingiverse.com/thing:2199907. To create the physical model, a ventricle-shaped structure was 3D printed using water-soluble polyvinyl alcohol. This model was embedded in PDMS and cured, after which it was dissolved in a shaking beaker filled with continuously heated water (35 °C) for three days. FEA simulation was performed using COMSOL Multiphysics 6.1 (Burlington, MA, USA). A 3D ventricle model was imported, and a simplified cylindrical DBS electrode model was created within the ventricle to replicate the implantation condition.

## Supplementary information

Below is the link to the electronic supplementary material.

Supplementary Material 1

## Data Availability

No datasets were generated or analysed during the current study.
