# Supplementary material for: Neuroendoscopy-compatible neurostimulation catheter for minimally-invasive and multifunctional hypothalamic deep brain stimulation
Source: Biomed Microdevices. 2026 Mar 12;28(1):22. doi: 10.1007/s10544-026-00804-2 (PMC12982212; doi:10.1007/s10544-026-00804-2)
Supplement: Supplementary file 1 — Supplementary Material 1 [file 10544_2026_804_MOESM1_ESM.docx]

Supplementary information

**Neuroendoscopy-compatible electronic catheter for minimally-invasive hypothalamic deep brain stimulation**

Jae Young Park^1,2,3^, Juan C. Mesa^1,2,3^, Jongcheon Lim^1,2,3^, Sergio Ruiz Vega^1,2,3^, Deniz Eksioglu^1^, Albert Lee^1,4^, and Hyowon Lee^1,2,3*^

^1^ Weldon School of Biomedical Engineering, Purdue University, West Lafayette, IN, USA

^2^ Birck Nanotechnology Center, Purdue University, West Lafayette, IN, USA

^3^ Center for Implantable Devices, Purdue University, West Lafayette, IN, USA

^4^ Legacy Health, Portland OR, 98671, USA

^*^Corresponding Author E-mail: [hwlee@purdue.edu](mailto:hwlee@purdue.edu)


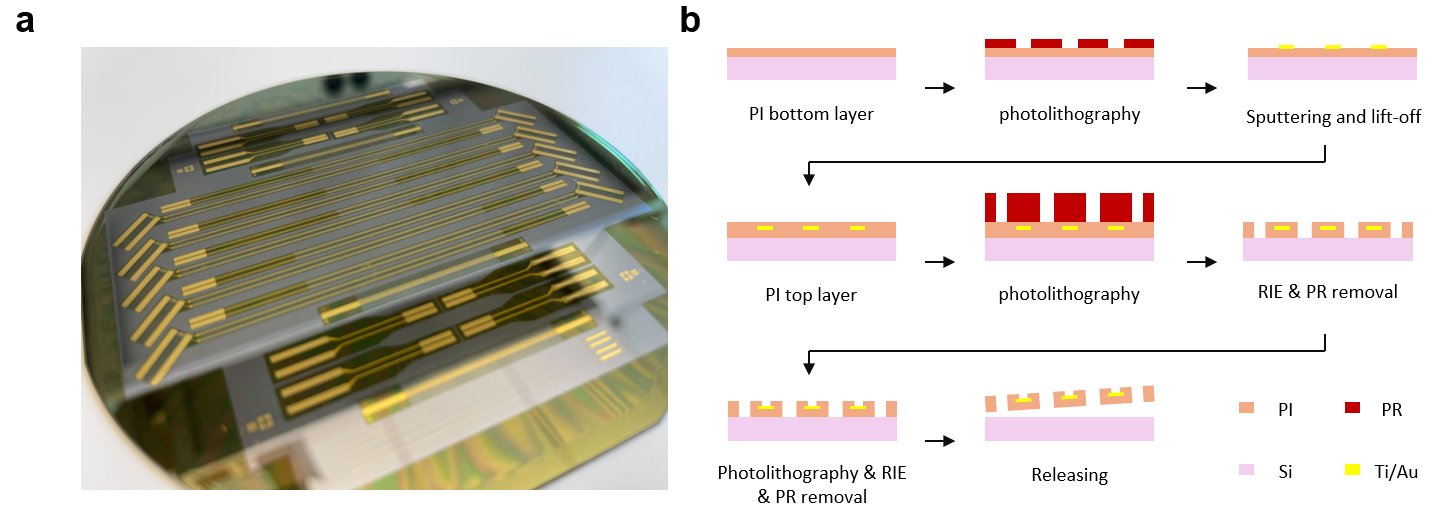


**Figure S1.** (a) thin-film electrodes for electronic catheter on a 4-inch wafer and (b) its microfabrication flow chart


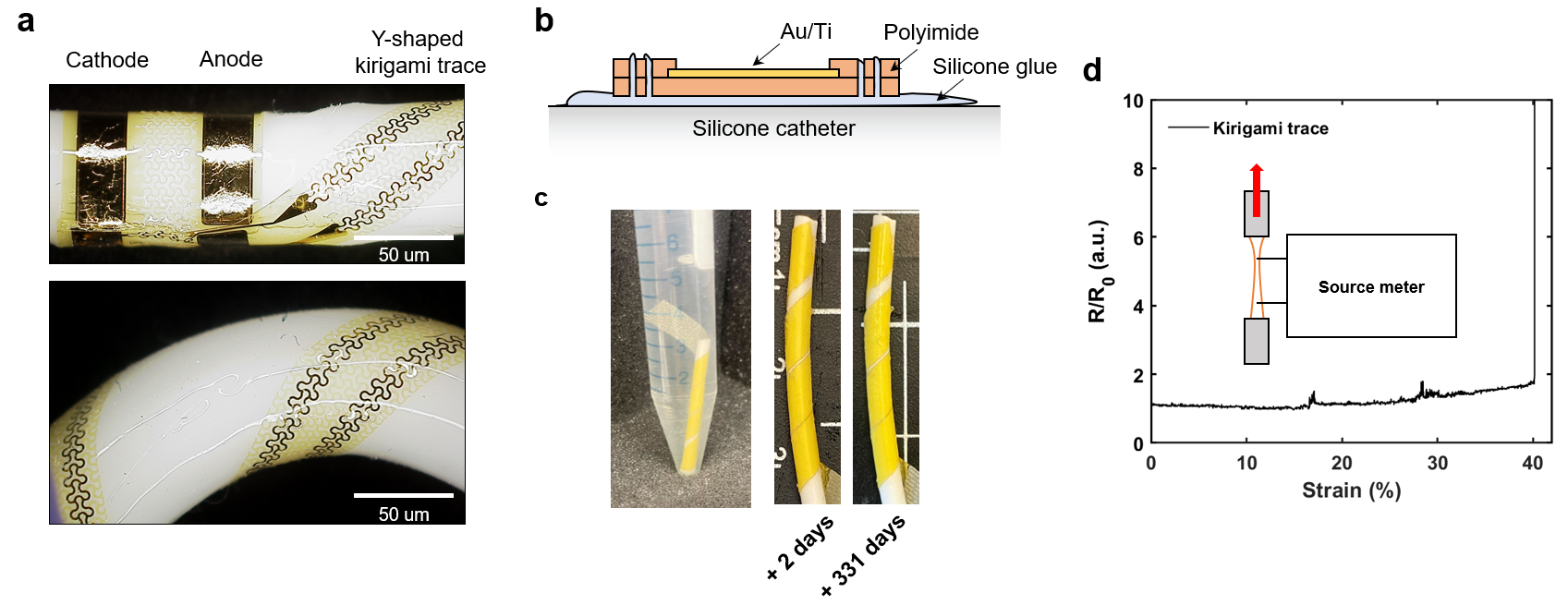
 **Figure S2.** (a) photographs of the electronic catheter (b) schematic cross-section illustrating the interface between the device and catheter (c) long-term stability analysis of the polyimide-to-catheter bonding in solution (d) electromechanical analysis of the kirigami-patterned trace.


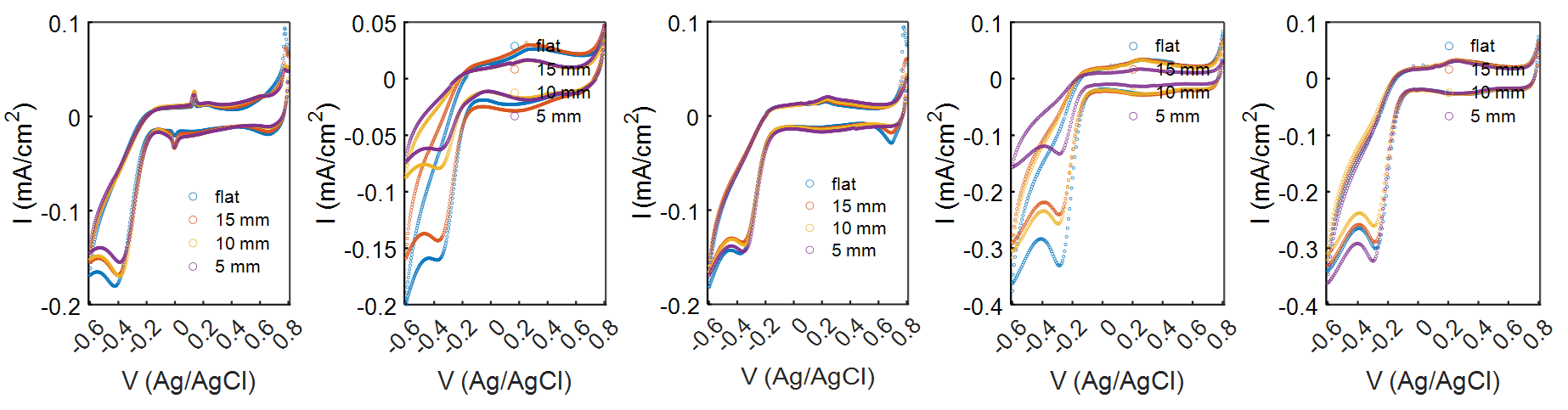


**Figure S3.** Bending radius-dependent cyclic voltammetry results of 5 samples.


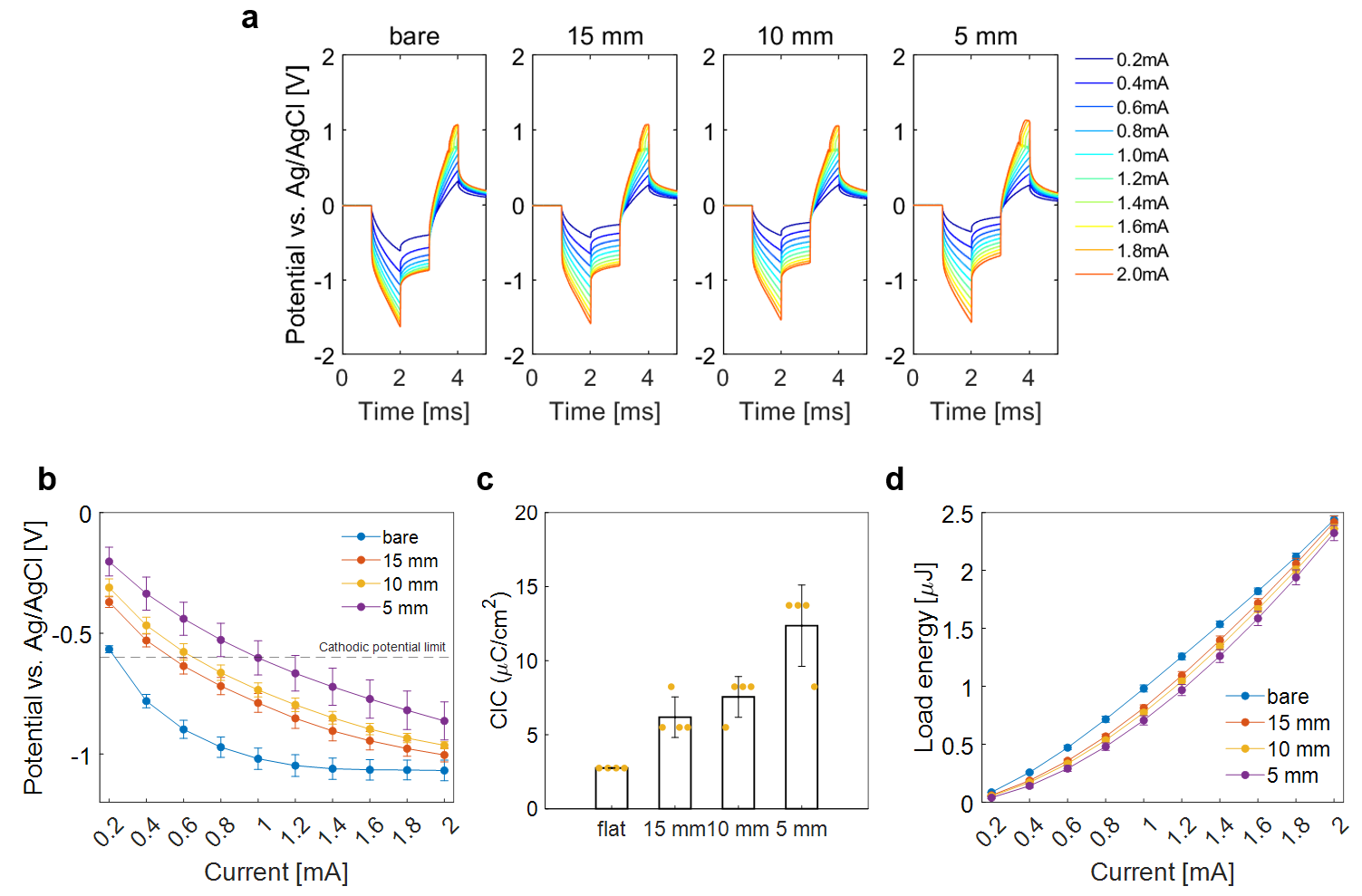


**Figure S4.** (a) voltage transient response (b) negative excursion potential (c) load energy according to bending radius.


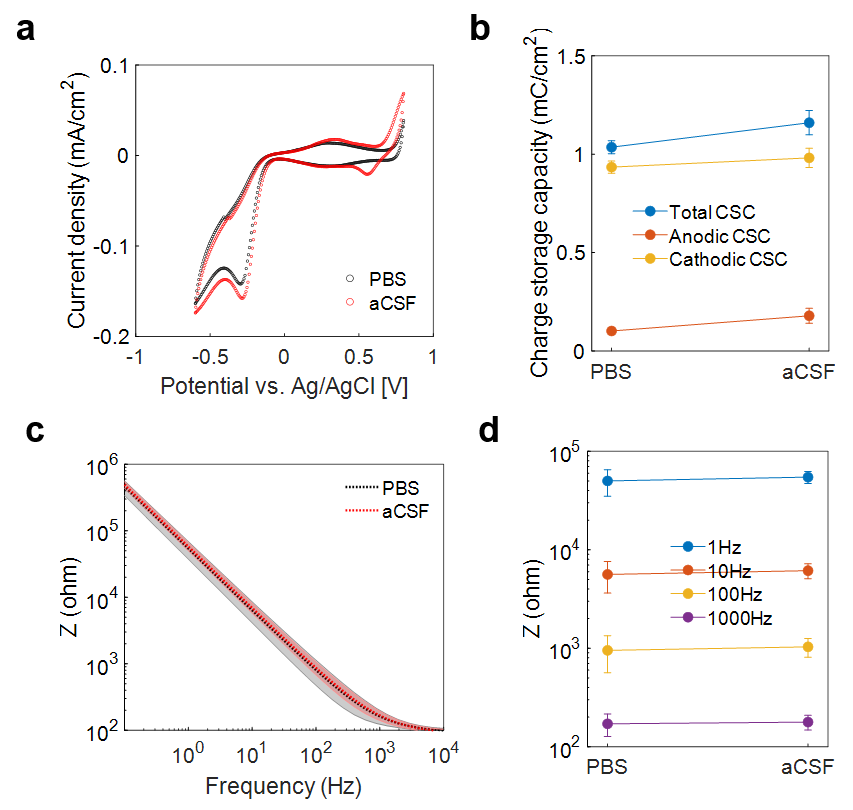


**Figure S5.** (a-b) cyclic voltammetry (c-d) electrochemical impedance spectroscopy of electronic catheter in PBS and aCSF.


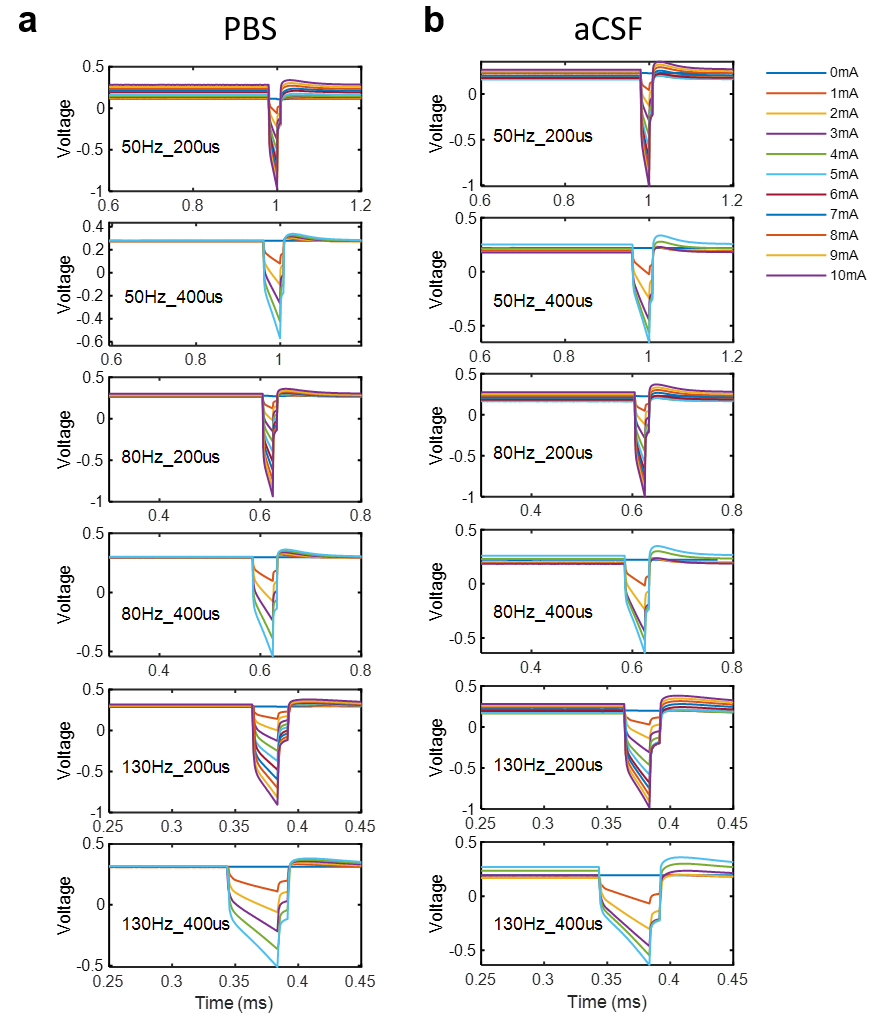


**Figure S6.** Stimulation analysis of electronic catheter in (a) PBS (b) aCSF.


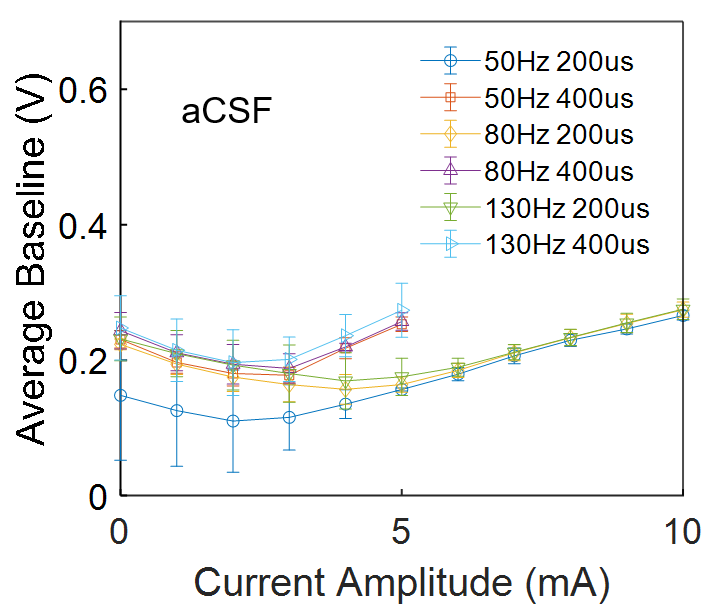


**Figure S7.** Baseline level according to current amplitude under various combinations of stimulation frequency and pulse width in a CSF.


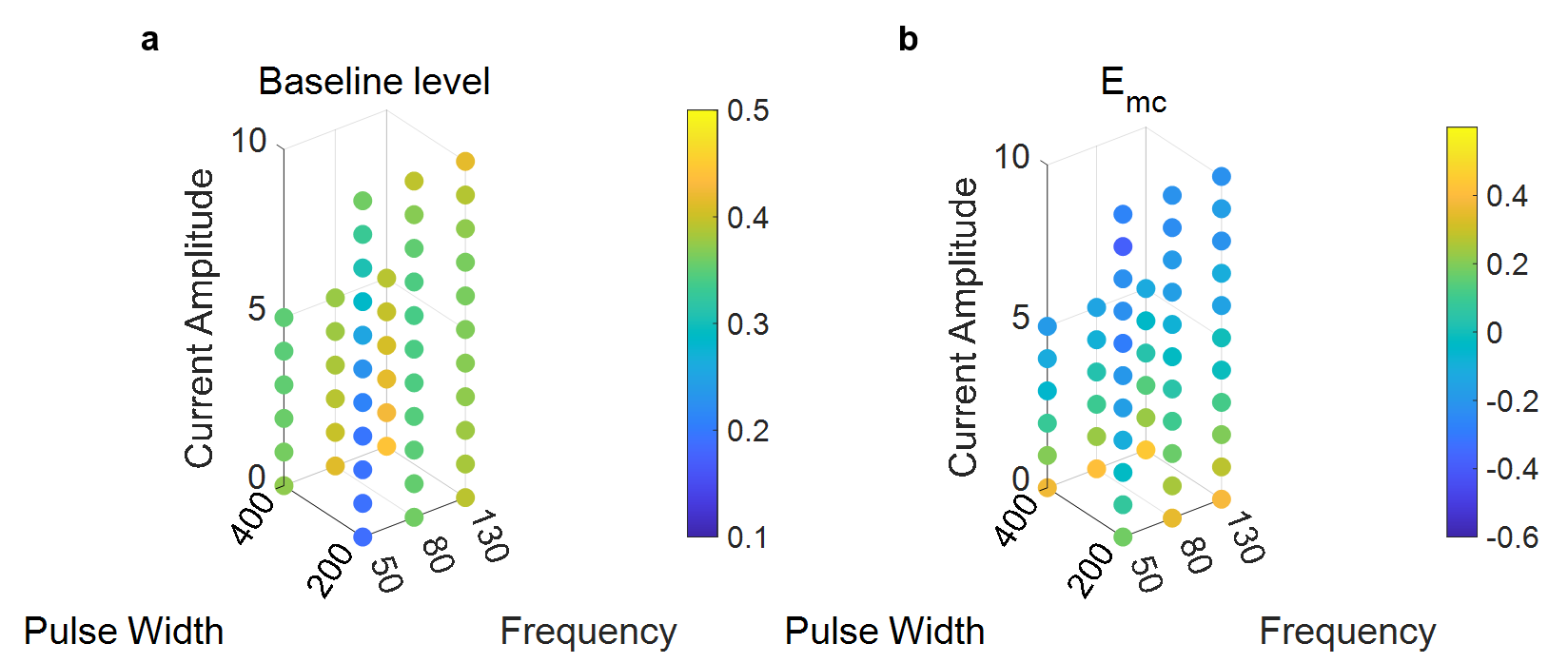


**Figure S8.** (a) baseline level and (b) negative excursion potential under various stimulation parameters in PBS


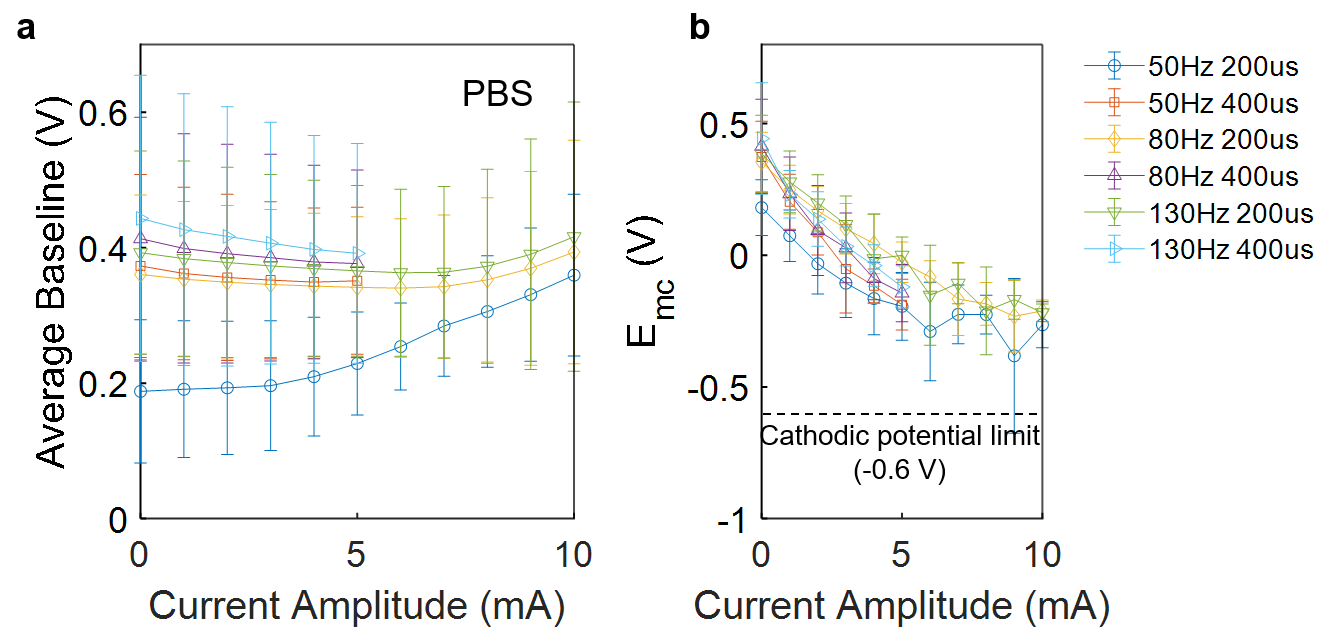


**Figure S9.** (a) baseline level and (b) negative excursion potential according to current amplitude under various combinations of stimulation frequency and pulse width in PBS.
